# Detection of carbapenem resistance and its attributable genes in *Acinetobacter baumannii* isolated from cardiac patients at a referral cardiac hospital of Kathmandu

**DOI:** 10.1186/s12866-025-04692-z

**Published:** 2026-01-09

**Authors:** Prashant B K, Sobita Khadka, Upendra Thapa Shrestha, Megha Raj Banjara

**Affiliations:** 1 Central Department of Microbiology, Tribhuvan University, Kirtipur, Kathmandu, Nepal; 2 Shahid Gangalal National Heart Centre, Bansbari, Kathmandu, Nepal

**Keywords:** Carbapenem resistance, MDR, Gene co-existence, *Acinetobacter baumannii*

## Abstract

**Objective:**

*Acinetobacter baumannii* has emerged as a major nosocomial pathogen due to its remarkable ability to develop resistance to multiple antibiotics including carbapenems. The objective of this study was to assess the carbapenem resistance and detect carbapenem-resistant genes in clinical isolates of *A. baumannii*.

**Methods:**

A cross-sectional study was conducted in Shahid Gangalal National Heart Centre, Kathmandu during February to September 2024. A total of 42 *A. baumannii* were isolated from different clinical specimens and identified. Antibiotic susceptibility test was performed by Kirby-Bauer disc diffusion method and carbapenemase production was assessed using modified carbapenem inactivation method and EDTA-carbapenem inactivation method. Carbapenem-resistant genes were detected through polymerase chain reaction. Among 1607 samples tested, 349 were culture positive for bacteria.

**Results:**

The prevalence of *A. baumannii* was 12% (42/349). Among the 42 *A. baumannii* isolates, 88.1% were resistant to carbapenems. Metallo-β-lactamase production was observed in 35.7% and multidrug resistance in 83.3% isolates. Resistance rates were highest against cefotaxime, cefepime and carbapenems. The *blaOXA-23* gene was detected in 69.1% of the isolates, *blaNDM-1* in 66.7%, and *blaVIM* in 14.3%, but none of the isolates harbored the *blaIMP*. Co-occurrence of *blaOXA-23* and *blaNDM-1* genes was detected in 20 (47.6%) isolates, *blaOXA-23*, *blaNDM-1* and *blaVIM* in 4 (9.5%) isolates, and *blaNDM-1* and *blaVIM* in 1 (2.4%) isolate.

**Conclusion:**

This study showed a high burden of carbapenems resistant and multi-drug resistant *A. baumannii*, likely contributed by the co-occurrence of carbapenem resistant genes. These findings provide valuable insights for clinical management and infection control of *A. baumannii.*

**Supplementary Information:**

The online version contains supplementary material available at 10.1186/s12866-025-04692-z.

## Introduction

*Acinetobacter baumannii* primarily causes hospital-acquired infections, but it can also occasionally lead to community-acquired infections such as endocarditis, peritonitis, cholangitis, and septic complications [1]. Its abilities to tolerate tough environments and quickly become resistant to various antibiotics allow it to persist on innate surfaces for extended periods, leading to the endemic presence of infections in patients [2]. *A. baumannii* has been considered as a critical priority pathogen due to its high morbidity and mortality rates among critically ill patients worldwide [3]. In this era of antibiotics, treating infections caused by *A. baumannii* is becoming increasingly difficult due to antimicrobial resistance [4]. The treatment protocol typically includes a combination of the beta-lactamase inhibitor sulbactam with broad-spectrum cephalosporins, quinolones, amikacin, doxycycline, minocycline, tigecycline, and polymyxins to combat these infections [5].

*A. baumannii* demonstrates a higher level of resistance to multiple major antibiotics classes, including carbapenems, beta-lactams, quinolones, and aminoglycosides. Carbapenem resistant *A. baumannii* (CRAB) has become a global threat for the clinical management [6]. Resistance to carbapenems is mediated by a variety of intrinsic carbapenem hydrolyzing genes [7]. The combination of two or more genes contributes not only to carbapenem resistance but also influences the organism’s overall antibiotic susceptibility. The major mechanisms for antimicrobial resistance (AMR) in *A. baumannii* are carbapenemase production, efflux pump, biofilm formation, decreased permeability, and the ability to pick up resistance genes via plasmids, especially in the hospital environment [8, 9].

Research has identified several genes responsible for carbapenem resistance. All strains of *A. baumannii* inherently possess the intrinsic carbapenemase *OXA-51-like* and is commonly employed as a molecular marker for species identification [10]. In addition to this intrinsic gene, *OXA*-type genes such as *blaOXA-23*,* blaOXA-24*, and *blaOXA-58*, have been identified in CRAB isolates [11, 12]. The presence of these genes, along with the production of metallo-β-lactamases (MBLs), significantly contributes to carbapenem resistance [11]. Metallo-β-lactamase such as NDM (New Delhi metallo-β-lactamase), VIM (verona integron-encoded metallo-β-lactamase) and IMP (imipenemase) are also critical contributor to resistance [13].

In Nepal, the misuse of antibiotics is prevalent, compounded by inadequate healthcare systems and poor infection control practices [14]. Timely detection of resistance genes is crucial for effective patient management, improved infection control measures and the development of new treatment strategies [12]. The scarcity of data on *A. baumannii* in Nepal poses significant barrier to designing effective, evidence-based treatment and infection control measures. With comparison to previous published studies from Nepal, this study focused on *A. baumannii* isolates from cardiac patients from referral level cardiac hospital of Kathmandu valley, assessed carbapenem resistance and its contributing genes, and explored the potential extensive antibiotic resistance due to multi-gene co-existence. The objective of this study was to determine the prevalence of carbapenem resistance, assess antibiotic susceptibility pattern and associated carbapenemase genes specifically *blaOXA-23*, *blaOXA-51*, *blaIMP*, *blaNDM-1*, and *blaVIM* in *A. baumannii* from clinical samples.

## Materials and methods

### Research design

This hospital-based cross-sectional study was conducted at a tertiary care Shahid Gangalal National Heart Centre (SGNHC) located in Kathmandu. The study was conducted during February to September, 2024. The bacteria isolated from the clinical specimens were collected and processed at the SGNHC microbiology and molecular laboratory. The bacteria identified as *A. baumannii* were further investigated for carbapenem resistance phenotypically and genotypically at Central Department of Microbiology, Tribhuvan University.

### Ethical approval

Ethical approval for the study was obtained from the Institutional Review Committee (IRC) of Shahid Gangalal National Heart Centre (SGNHC) Hospital (Ref. No.: 4-2024). The patients were informed of the study and samples and data were collected only after written informed consent.

### Study population and sample collection

The study population consisted of inpatients of SGNHC, including all age groups and both genders. A total of 1607 non-duplicated clinical specimens including wound swabs, pus, blood, urine, sputum, catheter tips, tissue, and body fluids from the patients of intensive care unit (ICU), critical care unit (CCU), and general ward (GW), were collected aseptically, in clean, leak-proof containers appropriate for each specimen type, following standard microbiological guidelines. The properly labeled sample with no visible signs of contamination, and having patients complete demographic data were accepted otherwise second sample was appealed. Patient samples with incomplete demographic information, unlabeled or duplicated samples, contaminated samples, and samples from OPD patients were excluded from the study.

### Culture of specimens

Sputum, urine, pus, and wound swabs and blood were inoculated in blood agar and MacConkey agar plates for microbial culture. Tissue specimens, pleural fluid, and endotracheal tubes underwent a preliminary enrichment in brain heart infusion (BHI) broth for two hours before being plated onto the media. Blood specimens collected under aseptic condition were diluted BHI broth in a ratio of 1:10 and thoroughly mixed to ensure homogeneity. The BHI broth was incubated at 37 °C for 96 h, with subculture transferred onto blood agar, MacConkey agar plates at 24 h intervals. Blood agar plates were incubated in a 5–10% CO₂ atmosphere for 24 h, in accordance with protocol outlined by Chesbrough (2006).

### Identification of *A. baumannii* and preservation

Bacteria that were coccobacilli morphology on Gram staining, catalase-positive, oxidase-negative, non-motile, indole-negative, citrate-positive, urease-negative, alkaline/alkaline reaction without H₂S production on Triple Sugar Iron (TSI) agar, and grew at temperatures between 37 °C and 44 °C were identified as *A. baumannii* [15]. Further, PCR of *blaOXA-51* gene, an intrinsic marker of *A. baumannii* was used to confirm it [10].

Pure culture of *A. baumannii* were preserved in tryptic soy broth (TSB) supplemented with 20% glycerol and stored at -80 °C [16].

### Antibiotic susceptibility test

Commercial antibiotic discs from HiMedia were used for antibiotic susceptibility test (AST) for all isolated organisms. The test was conducted using the Kirby Bauer disc diffusion method following the CLSI guidelines [17]. In case of tigecycline susceptibility test, break points of enterobacteriaceae was used. The isolates resistant to three or more than three different class of antibiotics was considered as multi-drug resistant (MDR) [18].

### Phenotypic detection of carbapenem resistance

All 42 *A. baumannii* isolates were tested for the presence of carbapenemase enzymes. The modified carbapenem inactivation method (mCIM) was employed to detect carbapenemase production in *A. baumannii* while the EDTA-modified carbapenem inactivation method (eCIM) was used alongside mCIM to differentiate metallo-β-lactamases from serine carbapenemases.

Briefly, a 10-µL loopful of *A. baumannii* was obtained from an overnight culture of BA plate and mixed into 2 mL of tryptic soy broth (TSB). The mixture was vortexed for 10–15 s and a10-µg meropenem disk was added to each tube using sterile forceps ensuring that the entire disk was fully immersed in the suspension. Incubation was carried out at 37 °C for 4 h ± 15 min. Meanwhile, 0.5 McFarland turbidity solution of *E. coli* ATCC 25922 was prepared in NB or saline using the colony suspension technique. MHA plate was then introduced with *E. coli* ATCC 25922 following standard procedures. The plates were allowed to dry for 3–10 min before the meropenem disks were added. The meropenem disk from each TSB meropenem disk suspension was slowly extracted using a 10-µL loop. The flat side of the loop was placed against the edge of the disk to leverage surface tension and extract the disk from the liquid. The loop was then softly dragged and compressed along the inside edge of the tube to clear any excess inoculated media from the disk before placing it on the MHA plate inoculated with the meropenem-susceptible *E. coli* ATCC 25922 indicator strain. Up to four disks were placed on a 90 mm MHA plate. The MHA plates were incubated at 37 °C for 18–24 h, after which the inhibition zones were analyzed according to standard disk diffusion methods [19].

The eCIM test was performed alongside with the mCIM test and was interpreted valid only when the mCIM produced a positive result [20]. To perform the eCIM, two tubes were prepared, each containing 2 mL of TSB and 10 µg imipenem disk. The mCIM procedure was applied to one tube, while the second tube, was designated for the eCIM, while 20 µl of EDTA were added. Both tubes are processed simultaneously and incubated at 37 °C for 2–3 h. After incubation, the meropenem disks from both tubes are transferred to a MHA plate inoculated with the *Escherichia coli* ATCC 25,922 strain. An inhibition zone of ≥ 7 mm or more around the imipenem-EDTA disk was interpreted as a positive eCIM result [21].

### DNA extraction and detection of carbapenem resistant *blaOXA-23*, *blaNDM-1*,* blaVIM*,* BlaIMP* and *blaOXA-51* genes

The isolated *A. baumannii* was maintained in tryptic soy broth (TSB) for DNA extraction. The DNA extraction was carried out using the boiling method, which involved heat treatment in the presence of NaOH.

Amplification of carbapenemase genes was performed using the primers and PCR conditions for *blaOXA-51* [22], *blaOXA-23* [21], *blaIMP*, *blaVIM* and *blaNDM-1* [23] genes.

The PCR-amplified products were analyzed through gel electrophoresis using a 1.5% agarose gel prepared in Tris-acetate-EDTA (TAE) buffer with 0.5 µL of ethidium bromide incorporated as nucleic acid stain. 2 µL of a 100 bp DNA ladder, 2 µL of a negative control, and 2 µL of each PCR amplicon sample were loaded into the wells of the gel and the separated products were analyzed.

### Quality control

A purity plate was utilized to confirm that the inoculum for biochemical tests originated from pure culture and to ensure that the tests were performed under aseptic conditions. All antibiotics discs and Mueller Hinton agar (MHA) were inspected for manufacture, expiration date, lot number and stored under appropriate conditions. Control strains of *Pseudomonas aeruginosa* (ATCC 27853) and *Escherichia coli* (ATCC 25922) were used to standardize the antimicrobial susceptibility testing (AST). Quality and sensitivity were ensured by maintaining the thickness of the MHA at 4 mm and by standardizing the organism inoculum to a 0.5 McFarland suspension during AST.

### Data analysis and interpretation

All the study data were entered into the worksheet of the Statistical Package for Social Science (SPSS) Software. The statistical analysis was done using SPSS, Microsoft Excel and python programming language. The data and results were presented using percentages, rates, and inferential statistics. p-values were calculated comparing resistance rates between *A. baumannii* with no or only one gene and two or more genes responsible for carbapenem resistance using chi-square test.

## Results

One thousand six hundred seven clinical samples were collected including 57% from male and 43% from female. Among 1607 clinical specimens, 21.7% (349) showed growth of bacteria. Out of the 349 total positive isolates, 12% (*n* = 42) were *A. baumannii*, 22.6% (*n* = 79) were *Klebsiella pneumoniae*, followed by 20.6% (*n* = 72) *Staphylococcus aureus*.

### Distribution of *A. baumannii* isolates across different clinical samples

**Fig. 1:**
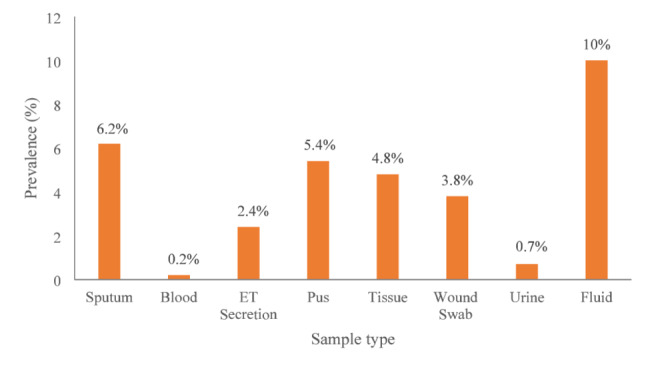
Distribution of *A. baumannii* isolates by sample type

Among the 42 *A. baumannii* isolates analyzed, 21 (50%) were obtained from the sputum samples, representing a prevalence of 6.2%. Four isolates were identified in both pus and urine samples, with a prevalence of 5.4% in pus and 0.7% in urine. Two isolates (3.8%) were obtained from wound swabs, while single isolates were identified in blood (0.2%), endotracheal (ET) secretions (2.4%), tissue (4.8%) and body fluid (10%) (Fig. 1).

Most *A. baumannii* isolates (40.5%) were recovered from patients admitted to the pediatric surgical intensive care unit (PSICU), with a higher frequency in male patients (13) than in females (4). In the adult surgical intensive care unit (ASICU), 23.8% of the isolates were identified, with higher number found in female patients (6) compared to males (4). The critical care unit (CCU) showed a relatively balanced distribution of isolates accounting for 21.4% of cases (females: 5; males: 4) while the general ward (GW) contributed 7.1% of total isolates exclusively recovered from female patients (3 cases). A total of 4.8% of the isolates were recovered from the operation theatre (OT), with one case each in male and female patients. In the pre-operative ward, 2.4% of the isolates were identified from male patients.

Among the 42 *A. baumannii* isolates, the highest prevalence was observed in infants aged 1–12 months (10 isolates; 23.8%), followed by patients aged 41–60 years (9 isolates; 21.4%) and those between 64 and 75 years (8 isolates; 19.1%). Slightly higher prevalence was seen in males (23 isolates; 54.8%) compared to females (19 isolates; 45.2%). Younger children contributed 5 (11.9%) cases, while the 19–40 age group was exclusively female (6 isolates; 14.3%). The lowest prevalence was seen in 12–18 years and 76 + years age groups, with 1 (2.4%) each contributing 1 case (2.4%).

### Antibiotic susceptibility pattern of *Acinetobacter baumannii*

High level of antibiotic resistance was observed in the *A. baumannii* isolates. Specifically, non-susceptibility to cefotaxime and cefepime was recorded in all tested isolates (100%). High percentage of isolates were resistant to ceftazidime (97.6%), ampicillin-sulbactam (85.7%), piperacillin-tazobactam (85.7%), imipenem (85.7%), and ciprofloxacin (88.1%). These isolates 42.9% showed sensitivity to tigecycline and 33.3% to amikacin. Lower sensitivity rates were recorded for gentamicin, cotrimoxazole and levofloxacin (28.6% for all three). The resistance rates against commonly used antibiotics were significantly higher among *A. baumannii* with two or more carbapenemase genes than with none or only one resistant gene (Table 1).

**Table 1:** Antibiotic resistance pattern of *A. baumannii*

Antibiotics	Resistant (%)	Difference in resistance		
		None or only one resistant gene (*n* = 18)	Two or more resistant gene (*n* = 24)	*p*-value
Ampicillin Sulbactam	36 (85.7)	12 (66.7)	24 (100.0)	0.004
Piperacillin-Tazobactam	36 (85.7)	14 (77.8)	22 (91.7)	0.375
Ceftazidime	41 (97.6)	17 (94.4)	24 (100.0)	0.429
Cefotaxime	42 (100)	18 (100.0)	24 (100.0)	-
Cefepime	42 (100)	18 (100.0)	24 (100.0)	-
Imipenem	36 (85.7)	12 (66.7)	24 (100.0)	0.004
Meropenem	37 (88.1)	13 (72.2)	24 (100.0)	0.010
Cotrimoxazole	30 (71.4)	10 (55.6)	20 (83.3)	0.049
Amikacin	28 (66.7)	6 (33.3)	22 (91.7)	< 0.001
Gentamicin	30 (71.4)	9 (50.0)	21 (87.5)	0.008
Tigecycline	24 (57.1)	5 (27.8)	19 (79.2)	0.001
Levofloxacin	30 (71.4)	6 (33.3)	24 (100.0)	< 0.001
Ciprofloxacin	36 (88.1)	12 (66.7)	24 (100.0)	0.004

### Prevalence of MDR and carbapenem-resistant *A. baumannii*

**Fig. 2:**
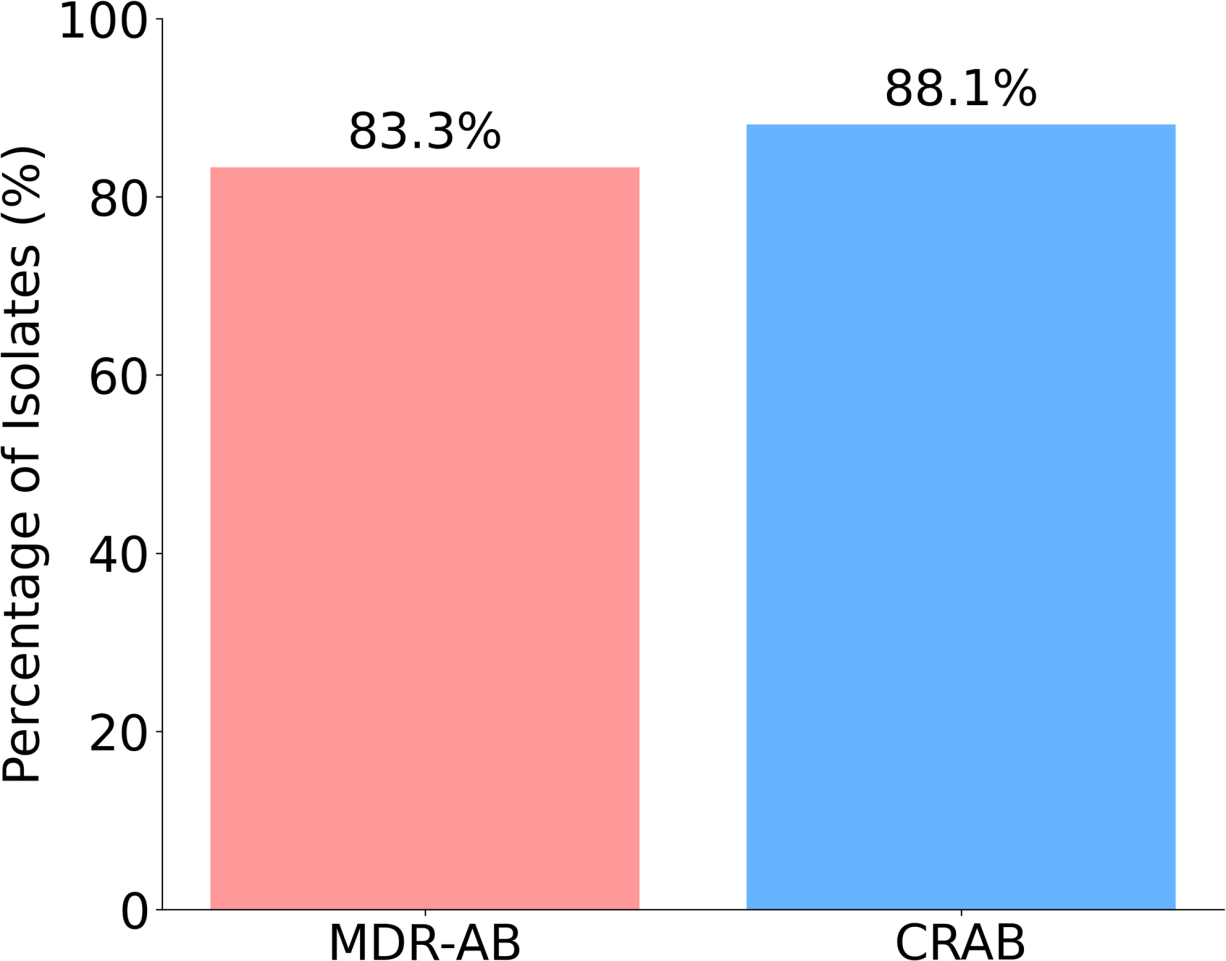
Prevalence of MDR and carbapenem-resistant *A. baumannii*

Among the 42 *A. baumannii* isolates, 83.3% were MDR-AB with an even more percentage of isolates (88.1%, *n* = 37) demonstrated resistant to carbapenems (Fig. 2). Further, 15 (35.7%) of the isolates were metallo-ß-lactamase producer as detected by eCIM method.

### Occurrence of carbapenem resistance genes in *A. baumannii* isolates

Among the 42 *A. baumannii* isolates phenotypically identified, the *blaOXA-51* gene was present in all isolates confirming them as *A. baumannii.* Five (5) carbapenem-sensitive isolates also carried the *blaOXA-51* gene. The *blaOXA-23* gene was detected in 69.1% of isolates, whereas *blaNDM-1* was found in 66.7% of isolates. Additionally, the *blaVIM* gene was detected in 14.3% of isolates, but none of the isolates harbored the *bla-IMP* gene. Among three *blaOXA-23*, *blaNDM-1* and *blaVIM* genes detected, *blaOXA-23* (78.4%) and *blaNDM-1* (73.0%) were common in carbapenemase producers. Among 5 carbapenemase non-producers, only one isolate had *blaNDM-1* gene (Table 2, Supplementary file 1).

**Table 2:** Prevalence of carbapenem resistance genes in *A. baumannii* isolates

Resistance gene	Carbapenemase producer (*n* = 37)	Carbapenemase non-producer (*n* = 5)
*blaOXA-51*	37 (100%)	5 (100.0%)
*blaOXA-23*	29 (78.4%)	0 (0.0%)
*blaNDM-1*	27 (73.0%)	1 (20.0%)
*blaIMP*	0 (0.0%)	0 (0.0%)
*blaVIM*	5 (13.5%)	1 (20.0%)

### Co-existence of *blaOXA-23* with MBL genes (*blaNDM-1* and *blaVIM*) in *A. baumannii*

**Fig. 3:**
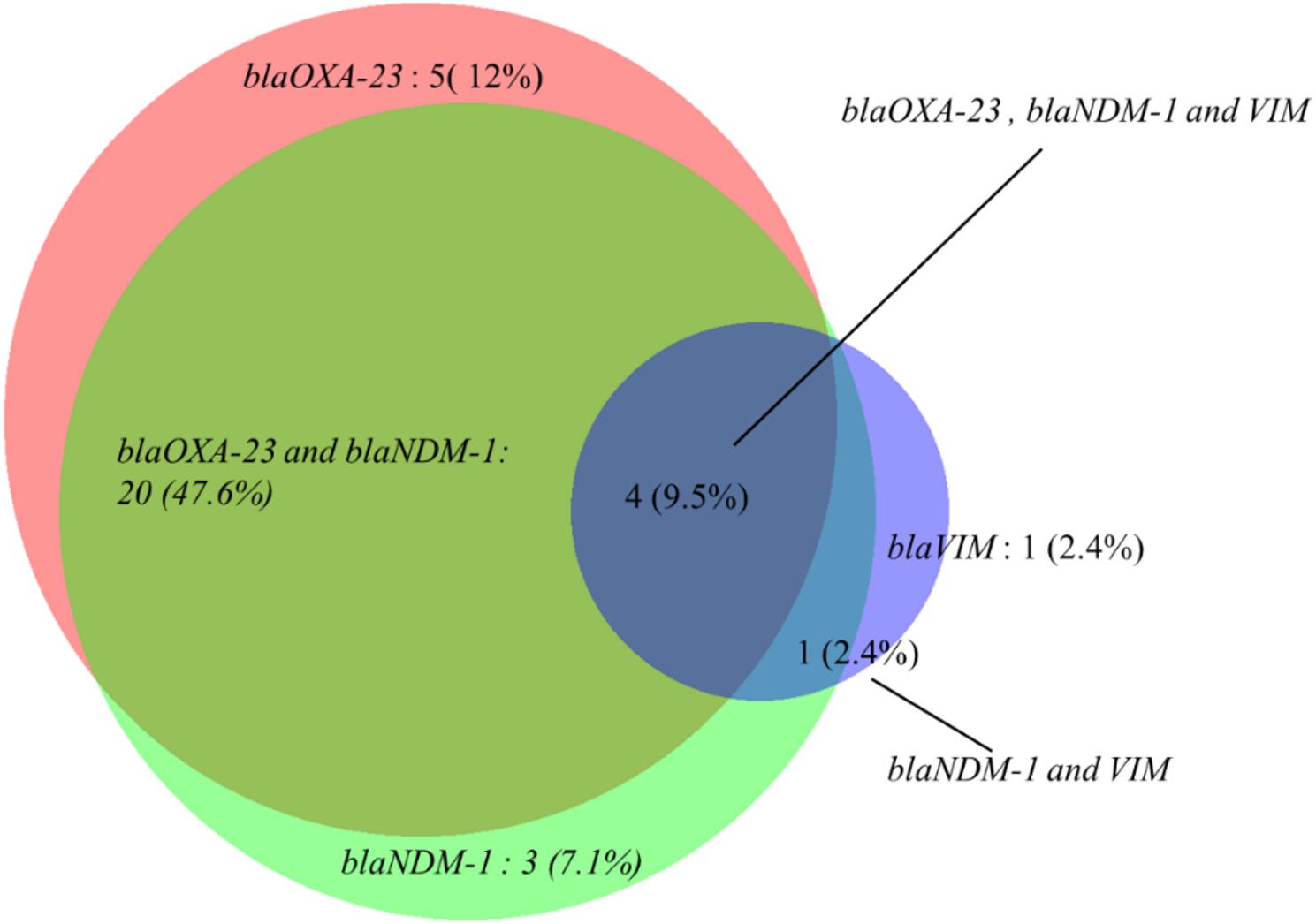
Co-existence of carbapenem resistance genes in *A. baumannii*

Co-carriage of *blaOXA-23* and *blaNDM-1* genes was detected in 20 isolates (47.6%). The combination of all three major carbapenemase genes (*blaOXA-23*, *blaNDM-1*, and *blaVIM*) was observed in four isolates (9.5%). Other specific gene combinations co-occurred at a low frequency; a single isolate (2.4%) carried both *blaNDM-1* and *blaVIM*. Most of the isolates lacked co-carriage of all three genes (Fig. 3).

## Discussion

We found that *A. baumannii* remains a significant nosocomial pathogen among cardiac patients in Nepal. In this study, the prevalence of *A. baumannii* among culture-positive clinical specimens was 12%, similar to previous studies in Nepal [24, 25]. There were varying prevalence rates of *A. baumannii* in clinical samples ranging from 4.3 to 15.7% in different clinical settings [26, 27]. Divergence in prevalence reports reflects variation in hospital antibiotic stewardship programs, patient’s admission patterns and sampling methods. Therefore, efforts to prevent infections by *A. baumannii* in health care settings are important.

The distribution of *A. baumannii* isolates across various specimen types highlights the predominance of *A. baumannii* in respiratory specimens, emphasizing its role in nosocomial respiratory infection. This finding is strongly consistent with established literature that identified respiratory and pus samples as the primary sources of *A. baumannii* isolates [26]. Previous study in Nepal also reported that majority of *A. baumannii* were from respiratory tract specimens, with the ICU being the most frequent site of isolation [28]. The organism’s capacity for biofilm formation on endotracheal tubing and its environmental resilience likely contribute to its high rate of recovery from the respiratory tract. Its recovery from pus, wound swabs and tissue samples aligns with its well-documented role in skin and soft tissue infections, particularly in immunocompromised patients [29]. The relatively low prevalence in urine may suggest that *A. baumannii* is not a primary uropathogen in our setting.

*A. baumannii* was distributed across various hospital departments, with 85.7% of isolates in critical care settings. These critical care settings present an ideal environment for the proliferation of MDR organisms such as *A. baumannii*, largely due to the vulnerable immunocompromised hosts, use of invasive medical devices, and antimicrobial therapy [30]. The detection of isolates from the operation theatre was concerning, as it points to direct inoculation and surgical site infections. The age-wise distribution revealed a bimodal distribution of *A. baumannii* infection, predominantly affecting neonates and elderly patients, who are immunocompromised and particularly vulnerable [31, 32].

This investigation showed an alarming level of antibiotic resistance profile in *A. baumannii* isolates, with a large proportion demonstrating resistance to widely practiced antibiotics. The most striking observation was the near-complete ineffectiveness of multiple classes of commonly used antibiotics. Out of 13 antibiotics tested, total resistance was noted for cefotaxime and cefepime. High resistance rates were also recorded for ceftazidime (97.6%). This broad resistance rate renders the cephalosporin class clinically ineffective. Additionally, high resistance rates were observed for piperacillin-tazobactam and fluoroquinolones like ciprofloxacin. Tigecycline resistance was also in high rate. We used break points of enterobacteriaceae for tigecycline to interpret the susceptibility test results of *A. baumannii* and this results should be interpreted cautiously. This extensive resistance significantly limits the available options for empirical treatment, compromising the efficacy of many standard broad-spectrum antibiotics. The greatest clinical concern was the high level of resistance to carbapenems, Widespread resistance to this class of antibiotics is a defining feature of difficult-to-treat *A. baumannii* and acquisition of carbapenem hydrolyzing enzymes, notably *OXA*-type carbapenemase [33].

We found that 83.3% of isolates were MDR, and 88.1% exhibited carbapenem resistance. This extreme resistance profile makes many antibiotic regimens, including those based on cephalosporins and fluoroquinolones, largely ineffective, severely restricting therapeutic options and presenting considerable challenges for clinicians.

Our findings of around two third prevalence of the *blaOXA-23* and MBL gene *blaNDM-1* genes confirms its status as the predominant *OXA*-type carbapenemase in *A. baumannii* isolates similar to other reports [27, 34, 35]. *blaOXA-51* gene was detected in all isolates of *A. baumannii* irrespective of phenotypic carbapenem susceptibility as *blaOXA-51* is an intrinsic species marker and does not independently confer carbapenem resistance [10]. The South Asian region has been recognized as the epicenter of the *blaNDM-1* outbreak and its widespread establishment in *A. baumannii* populations in Nepal has been previously reported [36, 37]. Moreover, identification of *blaVIM* genes adds to diversifies circulating MBLs, reflecting trends reported in other regional studies [13].

The co-carriage of *blaOXA-23* and *blaNDM-1* in significant proportion of isolates provided evidence for the observed extreme resistance particularly against beta-lactam antibiotics [38]. The isolates harboring all three genes, *blaOXA-23*, *blaNDM-1*, *blaVIM* represent a bacteria of highest concern. In our study, we detected around one tenth isolates with triple gene combination of *blaOXA-23*, *blaNDM-1* and *blaVIM*, indicating the increasing complexity and variety of resistance mechanisms in clinical environments. We also found concordance of presence of resistant genes or combination of genes with phenotypic carbapenemase producers. This may reflect the gene transfer or spread of multidrug-resistant strains in the ICU and high-risk wards. The co-existence of *blaOXA-23* and MBL genes like *blaNDM-1* and *blaVIM* are increasingly reported [39–41]. Pattern of multiple resistance gene carriage co-harboring *blaOXA-23*, *blaVIM-2*, and *blaNDM-1* was documented in regional reports [42, 43]. Our study also aligns with these multiple studies, which confirmed that co-occurrence of *blaOXA-23* with metallo-β-lactamase determinants such as *blaNDM-1* and *blaVIM* were recognized features of *A. baumannii* lineages [44].

This study has some limitations. The study was conducted over only eight months in a single tertiary care hospital, which may limit the generalizability of the findings beyond the study hospital. The relatively small number of *A. baumannii* isolates and lack of MIC data limited the inferential analyses comparing resistance patterns between gene groups. The associations should be understood cautiously. We confirmed *A. baumanii* using phenotypic, biochemical characteristics and PCR of *blaOXA-51* gene. This approach may not fully exclude other members of the *Acinetobacter calcoaceticus–baumannii* complex and we acknowledged the lack of molecular species confirmation using *gyrB* or *rpoB* gene as a limitation. Additionally, we did not assess colistin susceptibility, and we could not detect all carbapenemase genes. Further, we did not collect data on some resistance determinants and clinical outcome data from the infected patients.

## Conclusion

This study highlights the growing prevalence of carbapenem-resistant *A. baumannii* (CRAB), especially strains carrying carbapenemase genes, in clinical settings. High resistance rates to critical antibiotics such as cefotaxime, cefepime and carbapenems, reflect narrowing therapeutic options for treating *A. baumannii* infections. *A. baumannii* infections are concentrated in the intensive care units and surgical units, and excessively affect pediatric and elderly patients, along with the frequent recovery from respiratory samples such as sputum and endotracheal secretions, emphasizing the critical care burden and respiratory tropism of *A. baumannii*. The high rates of carbapenem resistant genes and the coexistence of them likely contribute to the development of extensive drug resistance. Regular monitoring and antibiogram profiling of MDR-AB and CRAB in the clinical setting is essential for effective treatment of infections.

## Supplementary Information

Supplementary Material 1.

## Data Availability

The datasets generated and analyzed during the study are available in the manuscript.

## Abbreviations

EDTA: Ethylenediaminetetraacetic Acid
CRAB: Carbapenem Resistant *Acinetobacter baumannii*
MDR: Multi Drug Resistance
