# Supplementary material for: Detection of carbapenem resistance and its attributable genes in Acinetobacter baumannii isolated from cardiac patients at a referral cardiac hospital of Kathmandu
Source: BMC Microbiol. 2026 Jan 9;26:128. doi: 10.1186/s12866-025-04692-z (PMC12911273; doi:10.1186/s12866-025-04692-z)
Supplement: Supplementary file 1 — Supplementary Material 1. [file 12866_2025_4692_MOESM1_ESM.docx]

**Supplementary files**


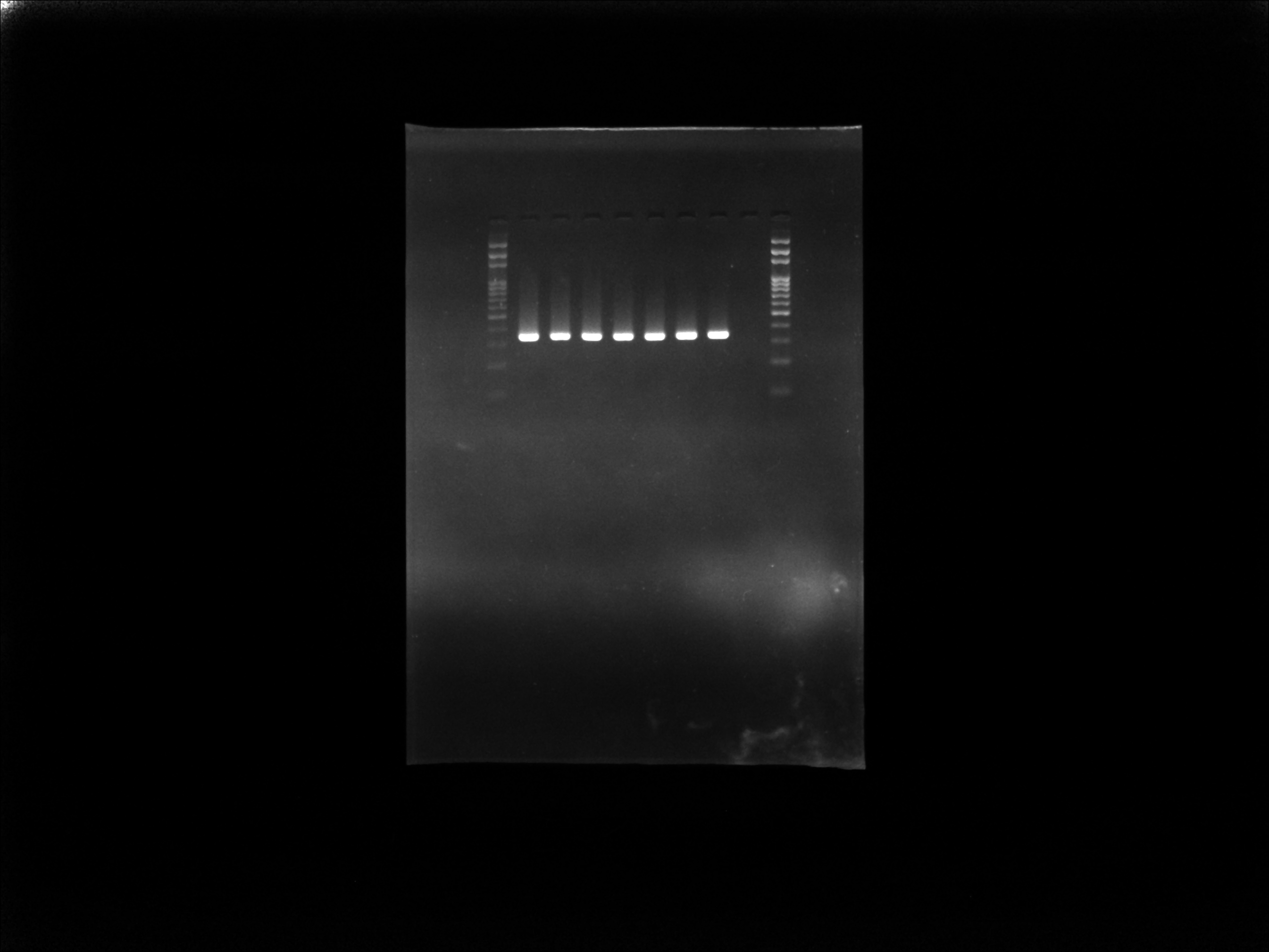


**Lane**

**353 bp**

***blaOXA-51***

**500 bp**

**800 bp**

**1000 bp**

**100 bp**

**300 bp**

**400 bp**

**500 bp**

**800 bp**

**1000 bp**

**200 bp**

**400 bp**

**100 bp**

**200 bp**

**300 bp**

**Ladder**

**1**

**6**

**5**

**Ladder**

**NCDDc**

**PCDD**

**4**

**3**

**2**

**Gel Photograph 1:** Gel electrophoresis of PCR amplicons of *blaOXA-51* (Lanes from left to right: marker, 1, 2, 3, 4, 5, 6- samples, PC-positive control, NC- negative control, marker)


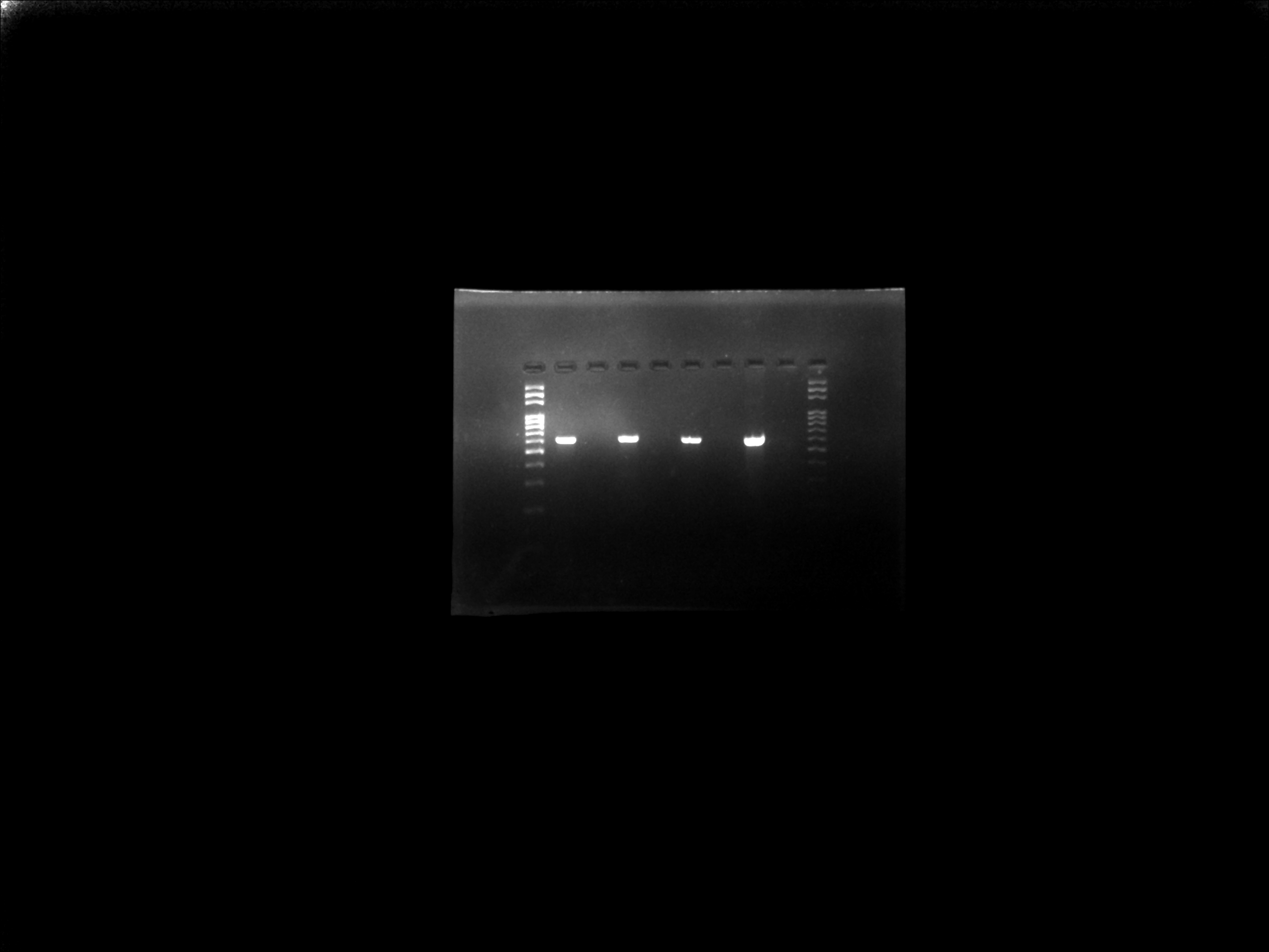


**Lane**

***blaOXA-23***

**501 bp**

**100 bp**

**200 bp**

**600 bp**

**500 bp**

**400 bp**

**300 bp**

**1000 bp**

**1000 bp**

**600 bp**

**500 bp**

**400 bp**

**300 bp**

**200 bp**

**100 bp**

**4**

**6**

**Ladder**

**1**

**3**

**5**

**2**

**PC**

**NC**

**Ladder**

**Gel Photograph 2:** Gel electrophoresis of PCR amplicons of *blaOXA-23* (Lanes from left to right: marker, 1, 2, 3, 4, 5, 6- samples, PC-positive control, NC- negative control, marker)
